# Phase II trial of ponatinib in patients with bevacizumab‐refractory glioblastoma

**DOI:** 10.1002/cam4.2505

**Published:** 2019-08-24

**Authors:** Eudocia Q. Lee, Alona Muzikansky, Dan G. Duda, Sarah Gaffey, Jorg Dietrich, Lakshmi Nayak, Ugonma N. Chukwueke, Rameen Beroukhim, Lisa Doherty, Caroline Kane Laub, Debra LaFrankie, Brittney Fontana, Jennifer Stefanik, Sandra Ruland, Victoria Caruso, Jennifer Bruno, Keith Ligon, David A. Reardon, Patrick Y. Wen

**Affiliations:** ^1^ Dana‐Farber/Brigham and Women's Cancer Center Boston Massachusetts; ^2^ Harvard Medical School Boston Massachusetts; ^3^ Massachusetts General Hospital Boston Massachusetts

**Keywords:** angiogenesis, bevacizumab‐refractory, FGFR, glioblastoma, VEGFR

## Abstract

**Background:**

Responses to bevacizumab in glioblastoma (GBM) are not durable. Plasma levels of basic fibroblast growth factor (bFGF) increase at the time of tumor progression. By targeting vascular endothelial growth factor receptor (VEGFR), platelet‐derived growth factor receptor, Src, and FGF receptor pathways, ponatinib may potentially help to overcome some of the putative mechanisms of adaptive resistance.

**Methods:**

We performed a phase II trial of ponatinib in patients with bevacizumab‐refractory GBM and variants. Adult patients with Karnofsky performance score (KPS) ≥60, measurable disease, and normal organ and marrow function received 45 mg ponatinib daily. No limit on the number of prior therapies but only one prior bevacizumab‐containing regimen was allowed. Primary endpoint was 3‐month progression‐free survival. Plasma biomarkers of angiogenesis and inflammation were evaluated before and after treatment.

**Results:**

The study closed after the first stage. Fifteen patients enrolled: median age 61 [27‐74]; median KPS 80 [70‐90]; median number of prior relapses 2 [2‐4]. Three‐month progression‐free survival rate was 0, median overall survival was 98 days [95% CI 56, 257], and median PFS was 28 days [95% CI 27, 30]. No responses were seen. The most common grade ≥3 adverse events included fatigue (n = 3), hypertension (2), and lipase elevation (2). Ponatinib treatment significantly increased plasma VEGF, soluble (s)VEGFR1, sVEGFR2, sTIE2, interferon gamma (IFNγ), tumor necrosis factor alpha (TNF‐α), interleukin (IL)‐6, IL‐8, and IL‐10 and decreased sVEGFR2.

**Conclusions:**

Ponatinib was associated with minimal activity in bevacizumab‐refractory GBM patients. Circulating biomarker data confirmed pharmacodynamic changes and suggested that resistance to ponatinib may be related to an increase in inflammatory cytokines.

## Graphical abstract

In this phase II trial, ponatinib was associated with minimal activity in bevacizumab‐refractory glioblastoma patients. Circulating biomarker data confirmed pharmacodynamic changes and suggested that resistance to ponatinib may be related to an increase in inflammatory cytokines.

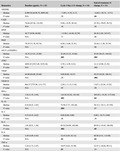

## 1 INTRODUCTION

Glioblastomas (GBMs) often develop resistance to treatment targeting vascular endothelial growth factor (VEGF) within months of starting therapy.^1^ Treatment options for tumors that progress despite bevacizumab are limited. In a phase II study, adding chemotherapy to bevacizumab in patients whose tumors already progressed on bevacizumab monotherapy was associated with little to no benefit.^2^ Mechanisms underlying resistance to antiangiogenesis agents in GBM are inadequately understood, but may include upregulation of alternative pro‐angiogenic pathways, vessel co‐option, increased invasiveness, and immune activation.^3^ Plasma levels of basic fibroblast growth factor (bFGF) increase in GBM patients treated with anti‐VEGF receptor (VEGFR) at the time of tumor progression, suggest that signaling by bFGF may play a role in resistance to antiangiogenesis agents.^4^, ^5^ In addition, preclinical evidence suggests that activation of Src family kinases plays an important role in glioma invasion.^6^, ^7^ Multikinase inhibitors targeting not only VEGFR but also fibroblast growth factor receptor (FGFR) and Src may potentially help overcome some of the putative mechanisms of resistance and result in increased antitumor effects.

Ponatinib is potent oral tyrosine kinase inhibitor that targets VEGFR, FGFR, Src, and platelet‐derived growth factor receptor (PDGFR). It is approved by the Food and Drug Administration in the United States for use in chronic myelogenous leukemia and Philadelphia chromosome‐positive acute lymphoblastic leukemia based on its ability to block BCR‐ABL (a mutation that is formed by the fusion of two genes, known as BCR and ABL) and other proteins. In U87 malignant glioma cell lines, ponatinib reduces cell viability, induces cell apoptosis, and suppresses migration and invasion.^8^ In a mouse xenograft model using U87MG, ponatinib reduced tumor growth in a dose‐dependent fashion by inducing cell apoptosis in vivo.^8^ We performed a single arm, open label, phase II and biomarker study of ponatinib in patients with bevacizumab‐refractory GBM.

## 2 MATERIALS AND METHODS

### 2.1 Patient eligibility

Eligible patients included adults (age ≥ 18 years old) with histologically confirmed GBM or variants who progressed following an anti‐VEGF(R) containing regimen. Any number of prior relapses on non–anti‐VEGF(R) containing regimens were allowed, although only one prior relapse on a bevacizumab or anti‐VEGF(R) containing regimen was allowed. Additional inclusion criteria included Karnofsky performance score (KPS) ≥60; adequate bone marrow, renal, and hepatic function; and measurable disease at baseline. Exclusion criteria included known coagulopathy, history of grade ≥3 hemorrhage within 30 days prior to study entry, poorly controlled diabetes defined as HgbA1c ≥7.0%, grade 3 ≥peripheral motor or sensory neuropathy, medications or substances that are moderate and strong inhibitors or inducers of CYP3A4, known Torsades de Pointes or QT prolongation, uncontrolled hypertriglyceridemia defined as triglycerides ≥450 mg/dL, history of acute pancreatitis, history of alcohol abuse, uncontrolled hypertension, and history of a clinically significant, uncontrolled, or active cardiovascular disease.

The study was approved by the institutional review board of Dana‐Farber/Harvard Cancer Center and conducted in accordance with institutional and federal guidelines for human investigations. All participants were informed of the investigational nature of this study and provided institutional review board‐approved informed consent before enrollment. The study was registered on http://clinicaltrials.gov (NCT02478164). The data that support the findings of this study are available on request from the corresponding author. The data are not publicly available due to privacy or ethical restrictions.

### 2.2 Treatment plan

Patients received ponatinib 45 mg daily in treatment cycles 28 days in length. For patients with stable disease (SD) or better at the end of Cycle 6, as determined by Response Assessment for Neuro‐Oncology criteria for high‐grade glioma,^9^ the dose of ponatinib was reduced to 30 mg daily due to the cumulative risk of vascular occlusive events observed in patients using ponatinib.^10^ Patients were evaluated every cycle. Brain magnetic resonance imaging (MRI) with contrast was obtained prior to the initiation of treatment and prior to every even numbered cycle including prior to cycle 2 (ie, after 28 days on therapy). Ponatinib was supplied by Takeda Oncology. Treatment continued until progressive disease or unacceptable toxicity.

The primary endpoint was 3‐month progression‐free survival (PFS3). Secondary objectives included radiographic response rate (RR), overall survival (OS), progression‐free survival (PFS), and safety. Survival analysis was based on Kaplan‐Meier estimates. Toxic effects were graded according to the National Cancer Institute Common Toxicity Criteria, version 4.0. In exploratory studies, we examined changes in plasma angiogenic biomarkers after therapy and their associations with outcomes.

### 2.3 Correlative studies

Blood collection for plasma angiogenic biomarkers was mandatory for all participants. Samples were collected at various time points: baseline (prior to starting therapy on Day 1), on Day 2 (prior to the second dose of ponatinib), on Day 1 of subsequent cycles, and off‐treatment. Plasma protein measurements were performed using multiplex array (Meso‐Scale Discovery) or standard ELISA kits (R&D Systems) in the Clinical Laboratory Improvement Amendments (CLIA)‐certified facility of the Steele Laboratories at Massachusetts General Hospital as previously described.^11^

Tumor genotyping was performed as part of routine clinical care in the CLIA‐certified facilities at Dana‐Farber Cancer Institute and/or Massachusetts General Hospital using methods as previously described.^12^, ^13^

### 2.4 Statistical analysis

The primary objective of the study was to determine the efficacy of ponatinib in participants with recurrent GBM who have progressed on a bevacizumab‐containing regimen as measured by PFS3. Three‐month progression‐free survival was chosen since agents with anti‐VEGFR activity may produce pseudoresponses, making response a less reliable endpoint. Based on retrospective data, PFS3 rate among recurrent GBM patients who received a second bevacizumab‐containing regimen after failing bevacizumab treatment once is 15%.^14^ This trial enrolled enough patients to discriminate between a 15% and 35% PFS3 rate. A Simon optimal two‐stage design was used to permit early termination of the study in case of futility. The first stage accrued 15 participants. If at least five or more of the first 15 participants achieved PFS3, accrual was increased to 12 more participants for a total of 27 participants. The study would have been declared successful if at least 10 or more out of 27 participants achieve PFS at 3 months. This design archives alpha error of 0.10 and beta error of 0.2. The probability of early termination if the drug was ineffective was 69%.

Plasma biomarker changes from baseline were expressed as percent change, reported as median with interquartile intervals. The significance of the change from baseline to Day 2 and end of treatment was assessed by the Wilcoxon Sign‐Rank test.

## 3 RESULTS

### 3.1 Patient characteristics, efficacy, and safety

We enrolled 15 patients with GBM or variants in Stage 1 (Table 1) between July 2015 and June 2017. Median age was 61 (range 27‐74) and median KPS was 80 (range 70‐90). The median number of prior therapies was 2 (range 2‐4) and the median time between last bevacizumab dose and first ponatinib dose was 34 days (range 20‐92). At the time of study enrollment, GBM with oligodendroglial features (GBMO) was a recognized GBM variant by the World Health Organization (WHO) Classification of Tumors. Upon central review, one patient had a GBMO with 1p/19q loss (which would be classified as an anaplastic oligodendroglioma by updated WHO 2016 criteria^15^).

**Table 1:** Patient characteristics

Patient characteristics	N = 15
Median age, y (range)	61 (27, 74)
Median KPS (range)	80 (70‐90)
Gender, female	4 (26.7%)
Race	
Caucasian	12 (86.7%)
Asian	1 (6.7%)
Multiracial	1 (6.7%)
Histology	
GBM	14 (93.3%)
GBM with oligodendroglial features	1 (6.7%)
IDH1/2 status	
IDH1/2 wild type by sequencing	9 (60%)
Negative for IDH1 R132H by immunohistochemistry	3 (20%)
Positive for IDH1 R132H by immunohistochemistry or sequencing	3 (20%)
No. prior Tx, median (range)	2 (2‐4)
Time between last bevacizumab dose and first ponatinib dose, median (range)	34 d (20‐92)

Abbreviations: GBM, glioblastoma; IDH, Isocitrate dehydrogenase; KPS, Karnofsky performance score.

As none of the patients achieved PFS3, the study was permanently closed after the first stage (Table 2). The longest time to progression observed was 84 days. Median PFS was 28 days [95% CI 27, 30] and median OS was 98 days [95% CI 56, 257]. There were no complete or partial responses seen and SD was the best response in two patients (13.7%). Toxicities on study were as expected for ponatinib with fatigue, increased lipase, and hypertension as the most common AEs (Table 3). Two patients were dose reduced, one due to grade 3 lipase and the other due to recurrent grade 2 diarrhea. One patient was taken off study due to unacceptable toxicity (grade 3 bullous dermatitis occurring during the first cycle). Another patient withdrew consent from study participation, also during the first cycle of treatment.

**Table 2:** Outcomes

Outcomes	N = 15
PFS3 rate, product limit estimate [95% CI]	0
PFS in days, median [95% CI]	28 [95% CI 27, 30]
OS in days, median [95% CI]	98 [95% CI 56, 257]
RR	
SD	2 (13.7%)
PD	10 (66.7%)
Unknown	3 (20%)

Abbreviations: OS, overall survival; PD, progressive disease; PFS, progression‐free survival; PFS3, 3‐month progression‐free survival; RR, radiographic response rate; SD, stable disease.

**Table 3:** Grade ≥3 toxicities possibly, probably, or definitely related to ponatinib

Toxicity (N, %)	Grade 3 (N = 15)	Grade 4 (N = 15)
ALT increased	1 (6.7%)	—
AST increased	1 (6.7%)	—
Bullous dermatitis	1 (6.7%)	—
Fatigue	3 (20%)	—
GGT increased	1 (6.7%)	—
Hypertension	2 (13.3%)	—
Lipase increased	2 (13.3%)	—
Lymphocyte decreased	1 (6.7%)	—

Abbreviations: ALT, alanine aminotransferase; AST, aspartate aminotransferase; GGT, Gamma‐glutamyl transferase.

### 3.2 Plasma biomarkers

The concentration of several plasma biomarkers of angiogenesis changed significantly after treatment with ponatinib (see Table 4). Ponatinib induced changes in pharmacodynamic biomarkers associated with anti‐VEGFR activity, such as decreased sVEGFR2 and increased sTIE2 and VEGF at the end of treatment. In addition, ponatinib treatment significantly and durably increased plasma concentration of sVEGFR1 and inflammatory cytokines including soluble IFN‐g, tumor necrosis factor alpha (TNF‐α), IL‐6, IL‐8, and IL‐10 (day 2 and end of treatment).

**Table 4:** Circulating plasma angiogenesis and inflammatory biomarkers

Biomarker	Baseline (pg/mL; N = 15)	Cycle 1 Day 2 (% change, N = 14)	End of treatment (% change, N = 11)
VEGFR2			
Median	6.909.20 [6430.70, 8099.20]	−3.89 [−6.70, 5.17]	−6.66 [−20.72, −0.71]
*P*‐value	N/A	.30	**.04**
CAIX			
Median	76.84 [47.94, 132.93]	9.56 [−8.39, 30.14]	25.78 [−39.07, 59.72]
*P*‐value	N/A	.10	.58
bFGF			
Median	46.77 [8.90, 68.90]	−13.96 [−44.98, 62.59]	48.36 [1.05, 243.67]
*P*‐value	N/A	.71	.06
PIGF			
Median	79.55 [71.78, 92.74]	8.28 [−4.68, 21.41]	10.44 [−1.30, 35.74]
*P*‐value	N/A	.09	.06
sFLT1 (sVEGFR1)			
Median	18.35 [13.41, 25.88]	22.40 [12.23, 43.66]	45.95 [36.23, 104.62]
*P*‐value	N/A	**.001**	**.001**
sTIE2			
Median	4029.25 [3513.49, 5235.16]	2.78 [−4.59, 9.21]	14.11 [5.90, 21.52]
*P*‐value	N/A	.50	.002
VEGF			
Median	64.00 [64.00, 85.68]	0.00 [0.00, 10.27]	49.23 [30.20, 100.51]
*P*‐value	N/A	.20	**.002**
VEGF‐D			
Median	864.37 [775.50, 1111.77]	2.20 [−11.13, 5.16]	‐5.67 [−23.54, 13.52]
*P*‐value	N/A	.95	.58
IFN‐γ			
Median	1.92 [1.35, 2.94]	110.36 [32.82, 183.48]	305.05 [−35.29, 1173.84]
*P*‐value	N/A	**.001**	**.04**
IL‐10			
Median	0.34 [0.23, 1.03]	75.56 [7.37, 182.28]	38.15 [−10.11, 417.95]
*P*‐value	N/A	**.004**	.07
IL‐2			
Median	0.35 [0.35, 0.62]	0.00 [0.00, 0.00]	0.00 [−10.75, 0.00]
*P*‐value	N/A	.88	.38
IL‐6			
Median	1.01 [0.51, 1.36]	65.52 [24.09, 102.68]	69.93 [−19.42, 346.59]
*P*‐value	N/A	**.002**	**.05**
IL‐8			
Median	4.49 [4.09, 6.44]	23.45 [4.84, 82.14]	80.56 [4.41, 114.69]
*P*‐value	N/A	**.02**	**.02**
TNF‐α			
Median	2.16 [1.73, 2.47]	18.97 [4.63, 53.50]	22.57 [−18.84, 89.11]
*P*‐value	N/A	**.01**	.24

Note

Medians and interquartile range for circulating plasma biomarker levels at baseline and percent changes after ponatinib treatment (significant changes are in bold, *P*‐values by Sign‐Rank test).

Abbreviations: bFGF, basic fibroblast growth factor; CAIX, Carbonic anhydrase IX; IFN‐γ, interferon gamma; IL, interleukin; PIGF, Placental growth factor; sFLT, soluble fms‐like tyrosine kinase; sTIE, soluble form of the Tie receptor; TNF‐α, tumor necrosis factor alpha; VEGF, vascular endothelial growth factor; VEGF‐D, vascular endothelial growth factor D; VEGFR, vascular endothelial growth factor receptor.

## 4 DISCUSSION

Ponatinib was associated with minimal activity in bevacizumab‐refractory GBM patients. The study closed early after the first stage for lack of efficacy. The preclinical models of ponatinib in GBM utilized only a single cell line (U87MG), which is suboptimal. Nevertheless, to date, no treatment has shown survival benefit in GBM patients who progress on bevacizumab. Evofosfamide plus bevacizumab,^16^, ^17^ dianhydrogalactitol (VAL‐083),^18^ and salvage re‐irradiation^19^, ^20^ have shown modest preliminary activity, although further studies are required to confirm their potential benefit.

Circulating biomarker data indicate that ponatinib has potent anti‐VEGFR activity as expected. Interestingly, Carbonic anhydrase IX (CAIX) (a biomarker of hypoxia) shows a nonsignificant trend for increase at day 2, but no change at the end of treatment. Increases in multiple inflammatory cytokines, including plasma soluble IFN‐g, TNF‐a, IL‐6, IL‐8, and IL‐10, at day 2 and end of treatment suggest immunomodulation after ponatinib treatment. Taken together, resistance to ponatinib may not be primarily related to hypoxia—induced by vascular rarefaction after VEGFR/FGFR inhibition—but rather to increased inflammatory cytokines. Indeed, some preclinical data suggest that certain immune cytokines may play a role in resistance to anti‐angiogenic therapy.^3^

Another possible explanation for ponatinib's lack of efficacy in bevacizumab‐refractory GBM is poor drug distribution into the tumor. Comparison between a heterotopic model (flank) and an orthotopic (intracranial) model of GBM demonstrated that a daily oral dose of ponatinib (30 mg/kg) was effective in reducing tumor growth of the flank tumor but not the intracranial tumor, which may be due to the regional differences in drug exposure across the intracranial tumor.^21^ Indeed, the total drug concentrations in the invasive rim of the intracranial tumor did not consistently exceed the in vitro cytotoxic concentration (IC_50_).^21^ There are limited data on the blood‐brain barrier penetration of ponatinib in humans.^22^

Rebound tumor progression has been reported following bevacizumab cessation.^23^ Whether rebound tumor progression due to cessation of bevacizumab contributed to poor RR and PFS3 and possible early discontinuation of treatment with ponatinib is unclear. The median time between last bevacizumab dose and first ponatinib dose on study was 34 days, and the first protocol brain MRI occurred after 28 days on ponatinib, so rebound tumor progression is conceivable. However, the poor median OS of patients on study suggests that these poor outcomes occurred regardless of potential rebound effects from bevacizumab discontinuation.

In addition to targeting VEGFR, PDGFR, and Src, ponatinib is also a potent pan‐FGFR inhibitor. Approximately 3.1% of patients with GBM harbor oncogenic chromosomal translocations that fuse the tyrosine kinase coding domains of FGFR genes to the transforming acidic coiled‐coil (TACC) coding domains.^24^ The FGFR‐TACC fusion protein demonstrates oncogenic activity and inhibition of this with use of an FGFR inhibitor has shown prolonged survival in mice with intracranial glioma harboring the FGFR3‐TACC3 fusion.^25^ Although we had hoped to study the effects of ponatinib in this molecular subgroup, none of the 12 patients enrolled on study with molecular testing by sequencing or array comparative genomic hybridization harbored evidence of FGFR‐TACC fusions.

In summary, ponatinib has limited efficacy in patients with bevacizumab‐resistant GBM. The circulating biomarker data suggest that immunomodulation may have played a role in resistance to treatment, although further studies are needed to clarify the interplay between angiogenesis and these immune cytokines. It is unclear if ponatinib could be beneficial in bevacizumab‐naïve patients or in patients whose GBM harbors a FGFR‐TACC fusion as neither of these populations were examined in this study. Given ponatinib's cumulative cardiovascular toxicity, potentially limited penetration across the blood‐brain barrier, and the recent drug development of selective brain penetrant FGFR inhibitors, further evaluation of ponatinib in GBMs with FGFR‐TACC fusion is not recommended.

## CONFLICT OF INTEREST

RB: Research support from Novartis; Owns equity in Ampressa; DGD: Consultant fees from Bayer, Tilos, twoXAR, and BMS; research grants from Bayer, Merrimack, Leap, Exelixis and BMS; JD: Consultant for Blue Earth Diagnostics and Royalties from UpToDate (Wolters Kluwer); EQL: Consulting from Eli Lilly; Royalties from to UpToDate, Inc (Wolters Kluwer) and MedLink, Inc; KLL: Consulting from BMS, Travera, Integragen, and Rarecyte. Research support to DFCI from BMS, Amgen, Lilly, Tragara, Plexxikon, Deciphera; LN: Consulting from BMS; DAR: Research support from Acerta Phamaceuticals; Agenus; Celldex; EMD Serono; Incyte; Inovio; Midatech; Omniox; Tragara. Advisory/consulting: Abbvie; Advantagene; Agenus; Bristol‐Myers Squibb; Celldex; DelMar; EMD Serono; Genentech/Roche; Inovio; Merck; Merck KGaA; Monteris; Novocure; Oncorus; Oxigene; Regeneron; Stemline; Taiho Oncology, Inc; PYW: Research support from Agios, Astra Zeneca, Beigene, Eli Lily, Genentech/Roche, Karyopharm, Kazia, MediciNova, Merck, Novartis, Oncoceutics, Sanofi‐Aventis, VBI Vaccines. Participated on advisory boards for Abbvie, Agios, Astra Zeneca, Blue Earth Diagnostics, Eli Lilly, Genentech/Roche, Karyopharm, Kiyatec, Puma, Vascular Biogenics, Taiho, Deciphera, VBI Vaccines, Tocagen. Speaker for Merck, Prime Oncology. The remainder of the coauthors declare that they have no conflict of interest.

## AUTHOR CONTRIBUTIONS

EQL, DGD, PYW have contributed to the concept and design of the study. EQL, JD, LN, UNC, RB, LD, CKL, DL, BF, JS, SR, VC, JB, DAR, PYW have contributed to the implementation of the data. EQL, AM, DGD, SG, PYW have contributed to the data analysis and interpretation. All authors were involved in the writing of the manuscript at draft and any revision stages and have read and approved the final version.

## ETHICAL APPROVAL

All procedures performed in studies involving human participants were in accordance with the ethical standards of the institutional and/or national research committee (include name of committee + reference number) and with the 1964 Helsinki Declaration and its later amendments or comparable ethical standards.

## Data Availability

The data that support the findings of this study are available on request from the corresponding author. The data are not publicly available due to privacy or ethical restrictions.
